# The safety of agomelatine in standard medical practice in depressed patients: A 26‐week international multicentre cohort study

**DOI:** 10.1002/hup.2759

**Published:** 2020-09-25

**Authors:** Philip Gorwood, Jacques Benichou, Nicholas Moore, Enric Álvarez Martínez, Joost Mertens, Eugenio Aguglia, Maria‐Luisa Figueira, Peter Falkai, Valérie Olivier, Marine Wattez, Françoise Picarel‐Blanchot, Christian de Bodinat

**Affiliations:** ^1^ GHU Paris Psychiatrie et Neurosciences (CMME, Hôpital Sainte‐Anne) Université de Paris & INSERM U1266 Paris France; ^2^ Centre Hospitalier Universitaire de Rouen Unité de Biostatistiques Rouen France; ^3^ Bordeaux PharmacoEpi CIC Bordeaux CIC1401 INSERM U1219 Hôpital Pellegrin Bordeaux France; ^4^ Hospital de Sant Pau Universitat Autònoma de Barcelona Institut de Recerca Biomedica Sant Pau Barcelone Spain; ^5^ De Velse GGZ Velsen‐Zuid The Netherlands; ^6^ Clinica Psichiatrica AOU Policlinico Vittorio‐Emanuele Catania Italia; ^7^ Faculty of Medicine University of Lisbon Lisbon Portugal; ^8^ Department of Psychiatry University of Munich Munchen Germany; ^9^ Institut de Recherches Internationales Servier (IRIS) Suresnes France

**Keywords:** agomelatine, liver acceptability, observational, safety‐medical practice, skin events

## Abstract

**Objective:**

The present observational cohort study documented the safety of agomelatine in current medical practice in out‐patients suffering from major depressive disorder.

**Method:**

The 6‐month evolution of agomelatine‐treated patients was assessed with a focus on safety (emergent adverse events, liver acceptability), severity of depression using the Clinical Global Impression Severity (CGI‐S) score, and functioning measured by the Sheehan Disability Scale (SDS).

**Results:**

A total of 8453 depressed patients from 761 centres in 6 countries were analysed (female: 67.7%; mean age: 49.1 ± 14.8 years). Adverse events reported were in accordance with the known safety profile of agomelatine. Cutaneous events were reported in 1.7% of the patients and increased hepatic transaminases values were reported in 0.9 % of the patients. The incidence of events related to suicide/self‐injury was 1.0%. Two completed suicides, not related to the study drug, were reported. CGI‐S total scores and SDS sub‐scores improved and numbers of days lost or underproductive decreased over the treatment period.

**Conclusions:**

In standard medical practice, agomelatine treatment was associated with a low incidence of side effects. No unexpected events were reported. A decrease in the severity of the depressive episode and improved functioning were observed.

**Trial registration name:**

Observational cohort study to evaluate the safety of agomelatine in standard medical practice in depressed patients. A prospective, observational (non‐interventional), international, multicentre cohort study.

**Trial registration number:**

ISRCTN53570733

## 1 INTRODUCTION

Major depressive disorder (MDD) is the most common psychiatric disorder worldwide, with a lifetime prevalence of approximately 13% (Alonso et al., 2004; Bromet et al., 2011; Ferrari et al., 2013), associated with marked morbidity and premature mortality (Chesney, Goodwin, & Fazel, 2014). The antidepressant agomelatine (Valdoxan^®^) is a melatonergic MT1/MT2 receptor agonist with serotonin 5‐HT2C receptor antagonist activity (Guardiola‐Lemaitre et al., 2014). With this unique mechanism of action, agomelatine has demonstrated a range of properties that suggest it could offer advantages over current treatments for MDD (Kennedy & Rizvi, 2010). The antidepressant efficacy of agomelatine has been demonstrated at doses of 25–50 mg in the treatment of the full range of depressive symptoms in patients with moderate to severe MDD (de Bodinat et al., 2010) and agomelatine showed a similar efficacy to other available treatments (Taylor, Sparshatt, Varma, & Olofinjana, 2014).

Agomelatine is generally well tolerated and shows a different profile of adverse events compared to selective serotonin reuptake inhibitors (SSRIs) and serotonin‐norepinephrine reuptake inhibitor (SNRIs), with a more favourable profile on gastrointestinal, psychiatric, cutaneous and vascular systems (Kennedy & Rizvi, 2010). Agomelatine also preserves the integrity of sexual function (Montejo et al., 2010, 2015) and is not associated with discontinuation syndrome after abrupt treatment cessation (Montgomery, Kennedy, Burrows, Lejoyeux, & Hindmarch, 2004). Nevertheless, cases of liver transaminases increase (AST/ALT > 3 ULN in 1.25% on agomelatine 25 mg, 2.62% on 50 mg, 0.5% of patients on placebo—Summary of product characteristics (SmPC) have been reported in agomelatine‐treated patients and in post marketing settings only, rare cases of hepatic failure were observed; therefore, a liver monitoring scheme is required and the drug is contraindicated in patients with impaired liver function.

At time of the product launch, in 2009, incidences of skin emergent adverse events were similar in agomelatine and placebo groups (1.17 and 1.25 per 100 patient‐months respectively). Skin reactions were however considered as a potential risk to be further monitored, based on the report of two serious cases (erythema nodosum recovered under treatment; erythematous rash) on agomelatine treatment.

In parallel, in depressed patients, the risk of suicide being high (American Psychiatric Association, 2003) this risk must be monitored. As treatment proceeds, variations in depressive symptoms may be associated with fluctuations in suicide risk. This monitoring includes evaluation of the presence of suicidal ideation and behaviours (American Psychiatric Association, 2010).

The current cohort study was designed to evaluate, in conditions of standard medical practice, the safety of a treatment with agomelatine prescribed to depressed patients. A focus was made on hepatic disorders (hepatic events with or without clinical symptoms including increase of transaminases >3 ULN), skin events, and suicidality (suicidal ideations, behaviours, and acts).

## 2 METHODS

### 2.1 Patients and study design

This was a prospective, observational, international, multi‐centre cohort study conducted in depressed patients followed‐up in current medical practice for their current depressive episode. The study was conducted in 761 centres in 6 countries. The physicians were recruited firstly through hospitals, clinics or private practices, in the speciality of psychiatry, then in the general practice.

Inclusion criteria were: male or female patients, more than 18 years of age, initiating agomelatine treatment for their current depressive episode, having signed an informed consent and accepting to give a personal reference contact.

Prescription of agomelatine resulted from a normal clinical evaluation according to the physician's clinical judgement, based on each patient's clinical profile and in line with the SmPC, including contra‐indications, special warning and precautions for use. The decision to enter a patient in the study, after his/her agreement, was done after the clinical decision to prescribe agomelatine.

Patients were not enrolled if they participated in another study, if they had to stop a successful on‐going antidepressant treatment, if they wished to continue another antidepressant treatment in addition to agomelatine, or if they planned to move during the follow‐up period of the study. No exclusion criteria were defined on potential comorbidities.

Patients were followed during 26 weeks of treatment with the usual clinical follow‐up provided by the involved clinicians. In case of agomelatine discontinuation, an end‐of‐study visit was scheduled 2 weeks after agomelatine withdrawal to follow safety and withdrawal symptoms.

For each patient, the dose and the duration of treatment were individually decided by the participating physician according to their usual medical practice, based on the approved SmPC and patients' clinical profiles. The starting dose of agomelatine was 25 mg once daily. A dose increase from 25 to 50 mg/day could be decided by the investigator. Patients who reported a dose of 25 mg/day throughout their follow‐up were considered in the ‘agomelatine 25 mg’ subgroup; patients with at least one intake of 50 mg were considered in the ‘agomelatine 25–50 mg’ subgroup.

### 2.2 Measurements

Physical examination and vital signs (heart rate, blood pressure, and weight) were assessed according to the usual care practice at baseline, follow‐up and end‐of‐study visits. Emergent adverse events (EAEs), including skin events (SOC), were collected at each visit and were defined as events occurring after the first study drug intake and up to 30 days after the last intake.

The report of adverse event was spontaneous. Physicians had to make sure that any adverse event/reaction or worsening of depression that occurred during the study was recorded. In particular, exacerbation of symptoms related to depression, including anxiety or other isolated symptoms possibly related to lack of efficacy of the study treatment or to cessation of treatment were to be recorded.

Liver function tests had to be performed as recommended in the agomelatine SmPC. Liver acceptability was assessed on both biological hepatic parameters with a focus on cases of aspartate aminotransferase (AST) and/or alanine aminotransferase (ALT) >3 ULN (upper limit of normal), and adverse events in accordance with investigator's judgement, link or not to biological abnormalities. For any AST and/or ALT > 3x ULN or evocating signs of hepatotoxicity, an adverse event had to be reported. A clinical review of narratives of all patients presenting with potentially clinically significant transaminases elevations (AST or ALT > 3 ULN) was made by a Liver Safety Committee composed of four hepatologists and a medical internist in order to assess causality of the reported abnormalities.

Suicidality (suicide, suicide attempt, suicidal ideation and self‐injuries behaviour) was assessed through the analysis of adverse events and using the Mini‐International Neuropsychiatric Interview (M.I.N.I.) items all along the study. Reviewing and adjudication procedures were set up and, in cases of serious suicidal behaviour (completed suicide or suicide attempt), were supervised by an external independent expert.

At each visit (baseline, follow‐up and end‐of‐study visits), the investigator assessed the severity of depression using the Clinical Global Impression (CGI; Guy, 1976, pp. 217–222) severity of illness (CGI‐S), and the patients' functioning using the Sheehan Disability Scale (SDS; Sheehan, Harnett‐Sheehan, & Raj, 1996). No other psychometric assessment tool was used as it was a non‐interventional study.

### 2.3 Statistical analysis

Quantitative variables were described by number of valid data, mean value and standard deviation. For qualitative variables, absolute and relative frequency distributions were presented.

Statistical analysis was performed on SAS^®^ software, version 9.2.

The study was run in accordance with the principles stated in the Declaration of Helsinki, Finland, Good Pharmacoepidemiology Practices (ISPE, 2008), Ethical Guidelines for Epidemiological, Studies (Rose, 2009) and the applicable regulatory requirements.

## 3 RESULTS

### 3.1 Baseline data

The study involved 1205 physicians from 761 centres in France (*n* = 265), Germany (*n* = 168), Italy (*n* = 105), Netherlands (*n* = 22), Portugal (*n* = 47) and Spain (*n* = 154), from December 2009 to August 2014.

Psychiatrists recruited 60.1% of patients, while general practitioners recruited 39.9% of patients. Of the 8743 patients included, 8453 patients with a signed inform consent and a date of agomelatine first intake were included in the analysis.

Out of the patients excluded due to unavailable signed informed consent, 14 patients reported at least one adverse event and were thus included in the safety cohort, which therefore consisted of 8467 patients. Main baseline characteristics of patients are presented in Table 1a. Patients had been depressed for more than 9 years in average and had a mean number of two major depressive episodes, including the current one. Based on the M.I.N.I. suicidality items, 34.4% of patients had a suicidal risk, most (15.7%) at a low level, 7.8% at a moderate level and 7.3% at a severe level of risk. Almost half of the patients reported at least one concomitant disease at inclusion. The most frequently reported comorbidities were hypertension, hypercholesterolemia, hypothyroidism, anxiety and diabetes mellitus (Table 1b).

**TABLE 1a:** Baseline characteristics of patients

	Agomelatine cohort (*N* = 8453)
Age (mean ± SD; years)	49.1 ± 14.8
Male/female (%)	32.3/67.7
Body mass index (mean ± SD)	25.5 ± 4.9
Number depressive episodes (including current one; mean ± SD)	2.0 ± 1.9
Disease duration (mean ± SD; years)	9.2 ± 9.5
Current episode duration (mean ± SD; months)	6.7 ± 14.7
Concomitant diseases (%)	46.7
CGI severity of illness score (mean ± SD)	4.5 ± 0.9
SDS total score (mean ± SD)	16.4 ± 5.9
SDS work (mean ± SD)	6.0 ± 2.4
SDS social life (mean ± SD)	6.2 ± 2.1
SDS family life (mean ± SD)	6.0 ± 2.2
Days unproductive^a^ (mean ± SD)	4.1 ± 2.6
Days lost (mean ± SD)	2.8 ± 2.8

Abbreviations: CGI, Clinical Global Impression; SD, standard deviation; SDS, Sheehan Disability Scale.

^a^ Patients felt so impaired by symptoms that their activity at school or at work was reduced.

The mean CGI‐S was 4.5 ± 0.9 (median: 5, markedly ill) and according to the SDS, on average, the patients felt moderately disrupted by disease symptoms and those linked to potential side effects for those on antidepressant treatments for the 3 functional domains.

**TABLE 1b:** Concomitant diseases at selection (reported in at least 1.5% of MDD patients in the cohort)

PT	Agomelatinecohort (*N* = 8453)
All *n* (%)	3944 (46.7)
Hypertension *n* (%)	1197 (14.2)
Hypercholesterolaemia *n* (%)	445 (5.3)
Hypothyroidism *n* (%)	284 (3.4)
Anxiety *n* (%)	257 (3.0)
Diabetes mellitus *n* (%)	240 (2.8)
Osteoarthritis *n* (%)	227 (2.7)
Insomnia *n* (%)	173 (2.0)
Obesity *n* (%)	154 (1.8)
Asthma *n* (%)	144 (1.7)
Type 2 diabetes mellitus *n* (%)	137 (1.6)
Alcoholism *n* (%)	123 (1.5)
Osteoporosis *n* (%)	129 (1.5)

*Note*: *n*, number of patients with the described concomitant disease. Percentage = (*n*/*N*) × 100.

Abbreviation: MDD, major depressive disorder.

### 3.2 Patient disposition

Most patients (76.1%) were treated with agomelatine 25 mg, whereas 23.9% of patients were treated with agomelatine 25–50 mg. Patients treated with the 25–50 mg dose had more severe depressive disorders and a higher disability at baseline than patients treated with the 25 mg fixed dose (e.g., in terms of disease duration, number of depressive episodes, hospitalizations and suicidal risk; Table 1c).

The mean treatment duration was 5.8 ± 2.9 months; 60.1% of patients being treated with agomelatine for at least 6 months. Treatment duration was similar in patients treated with the 25 mg dose (5.7 ± 2.9 months) and patients treated with the 25–50 mg dose (6.3 ± 2.7 months).

**TABLE 1c:** Baseline characteristics of MDD patients in the cohort by dose of agomelatine

	Agomelatine 25 mg fixed (*N* = 6431)	Agomelatine 25–50 mg (*N* = 2022)
Duration of the disease (mean ± SD; years)	8.9 ± 9.4	10.1 ± 9.7
Number of depressive episodes (including present one; mean ± SD)	1.9 ± 1.8	2.3 ± 2.2
Patients previously hospitalized for MDE (%)	7.5	12.8
Patients with previous treatment for depression (within 12 months; %)	28.4	37.3
Suicidal risk (%)	16.1	23.6
Patients with previous suicide attempts (%)	7.3	13.2
Suicidality scale (MINI; %)	29.2	40.7
Days unproductive (mean ± SD)	4.0 ± 2.6	4.6 ± 2.5
Days lost (mean ± SD)	2.7 ± 2.7	3.1 ± 2.8

Abbreviations: MDD, major depressive disorder; MINI, Mini‐International Neuropsychiatric Interview; SD, standard deviation.

A total of 2978 of patients (35.2%) were withdrawn from the study. The main reasons were remission or marked improvement (12.4% of patients), adverse event (8.6%), lack of efficacy (7.2%) and non‐medical reason (3.9%); 67 patients (0.8%) did not come back for the end‐of‐study visit. Out of the patients withdrawn from the study, 2829 (95%) stopped agomelatine, including 786 patients (27.8%) who switched to another antidepressant treatment, as 5.0% of patients remained on agomelatine treatment.

During the study period, 67% of patients treated by agomelatine (65.3% in the 25 mg fixed group and 72.3% in the 25–50 mg group) received at least one psychotropic concomitant treatment, anxiolytics (26.2% of patients) being the most common.

## 4 SAFETY RESULTS

### 4.1 Adverse events

The percentages of patients who reported at least one emergent adverse event (EAE) on treatment was 27.8%. The most frequently affected system organ classes were psychiatric disorders (8.7%), nervous system disorders (6.9%), infections and infestations (5.6%), and gastrointestinal disorders (5.2%). The most frequent EAEs were headache, nausea, anxiety and insomnia (Table 2a **)**. EAEs were considered as serious in 5.1% of patients and led to study treatment withdrawal in 7.7% of patients, mainly due to psychiatric and nervous system disorders (Table 2b).

**TABLE 2a:** EAEs reported in at least 0.5% of patients overall and by dose (Agomelatine safety cohort)

Preferred term	All agomelatine doses (*N* = 8467)		25 mg fixed (*N* = 6439)		25–50 mg (*N* = 2028)	
	*n*	%	*n*	%	*n*	%
ALL	2351	27.8	1703	26.4	648	32.0
Depression	229	2.7	127	2.0	102	5.0
Headache	213	2.5	151	2.3	62	3.1
Nausea	175	2.1	146	2.3	29	1.4
Anxiety	155	1.8	112	1.7	43	2.1
Insomnia	140	1.7	100	1.6	40	2.0
Alanine aminotransferase increased	107	1.3	68	1.1	39	1.9
Dizziness	102	1.2	80	1.2	22	1.1
Restlessnes	65	0.8	44	0.7	21	1.0
Fatigue	65	0.8	38	0.6	27	1.3
Gamma‐glutamyltransferase increased	62	0.7	42	0.7	20	1.0
Aspartate aminotransferase increased	61	0.7	37	0.6	24	1.2
Suicidal ideation	56	0.7	31	0.5	25	1.2

*Note*: *n*, Number of patients with at least one EAE in a given level. Percentage (*n*/*N*)*100.

Abbreviation: EAE, emergent adverse events.

**TABLE 2b:** Type of emergent adverse events in the agomelatine safety cohort, per dose of agomelatine, and in the subset of patients aged ≥65 years

Type of adverse events*	Agomelatine safety cohort (*N* = 8467)	Agomelatine 25 mg (*N* = 6439)	Agomelatine 25–50 mg (*N* = 2028)	Patients ≥65 years (*N* = 1256)
Serious EAE *n* (%)	433 (5.1)	278 (4.3)	155 (7.6)	86 (6.8)
Severe EAE *n* (%)	293 (3.5)	194 (3.0)	99 (4.9)	55 (4.4)
EAE related to treatment *n* (%)	761 (9.0)	559 (8.7)	202 (10.0)	111 (8.8)
Withdrawal due to EAE *n* (%)	651 (7.7)	550 (8.5)	101 (5.0)	121 (9.6)

*Note*: *n*, Number of patients with at least one EAE of the described type. Percentage (*n*/*N*)*100.

Abbreviation: EAE, emergent adverse events.

During the study, 22 patients (0.3%) died, among them 16 were aged 65 or older. The most frequent causes were related to infections and infestations (6 patients; 27.3%), general disorders malignant neoplasms, and respiratory, thoracic and mediastinal disorders (5 patients each; 22.7%). A sudden death occurred 2 months after the first agomelatine intake in a paraplegic patient; a cardiac arrest occurred in a patient with a severe motoneuron disease. Two patients completed suicide (vide supra). No death was considered related to the study treatment neither by the investigator nor by the sponsor.

### 4.2 Hepatic disorders

Hepatic EAEs, whatever the presence, nature or level of biological abnormalities, were reported for a total of 196 out of 8467 patients (2.3%).

The incidence was 2.0% with the 25 mg fixed dose and 3.3% with the 25–50 mg dose. The most frequent EAE was ‘ALT increased’ (1.3% of patients). ‘GGT increased’ and ‘AST increased’ were also reported (0.7% each).

Of note, among the 196 patients with emergent adverse events related to hepatic disorders, 139 (71%) did not present with transaminases increases >3x ULN.

Emergent hepatic disorders led to study drug withdrawal in 72 patients (36.7% of patients with events, i.e., 0.9% of the Agomelatine Safety Cohort). In about half of the patients with emergent hepatic disorders (99/196, 50.5%, i.e., 1.2% of the Agomelatine Safety Cohort) these EAEs were considered as treatment related by the investigators. All hepatic EAEs considered as study treatment‐related by the investigators were listed in the Reference Safety Information except one hepatic steatosis and 3 liver disorders (1 liver intolerance, and 2 minor increases in liver values of which one was presumably preexisting fatty liver).

Biological abnormalities were evaluated in patients with at least one post‐baseline value of ALT or AST, that is, 6687 patients (79.0% of the cohort) of whom 57 (0.9%) had at least one AST and/or ALT > 3 ULN (Table 3). Among those 57 patients, 54.4% had values between 3 ULN and 5 ULN, 28.1% had values between 5 ULN and 10 ULN, and 17.5% had values >10 ULN. Agomelatine was discontinued in 48 patients, temporarily interrupted in 3 patients (according to SmPC), and the dose was reduced in 2 patients, the study drug being maintained in 4 patients of whom 3 recovered on treatment. At the last available laboratory test, 37 out of 57 patients (64.9%) with transaminases values >3 ULN recovered, 8 patients (14.0%) were recovering between 1 and 2 ULN, 4 patients (7.0%) were recovering between 2 ULN and 3 ULN, and 6 patients (10.5%) did not recover. These transaminases increase occurred mainly within the first 3 months of treatment for 36 out of 57 patients. Among the ten patients who had transaminases value >10 ULN (7 patients on agomelatine 25 mg, 3 on agomelatine 25–50 mg), 5 cases occurred in a context of biliary colic associated with concomitant increase in total bilirubin above 2 ULN (2 patients), alcohol intoxication (2 patients), or intake of concomitant hepatotoxic treatment (1 patient). After reviewing of the 10 cases by the Liver Safety Committee, to assess the causality of the abnormalities, 3 cases were considered as probably related to agomelatine, 3 possibly related to agomelatine, 1 unlikely related to agomelatine, and 3 not related to agomelatine. All cases recovered.

**TABLE 3:** Emergent abnormal AST and/or ALT value (>3 ULN) in the safety cohort, per dose of agomelatine, and in the subset of patients aged ≥65 years

Abnormal values Classes	Safety Cohort (*N* = 6687)	Agomelatine 25 mg (*N* = 4923)	Agomelatine 25–50 mg (*N* = 1764)	Patients ≥65 years (*N* = 1033)
All *n* (%)	57 (0.9)	39 (0.8)	18 (1.0)	10 (1.0)
]3–5 ULN] *n* (%)	31 (0.5)	23 (0.5)	8 (0.5)	5 (0.5)
]5–10 ULN] *n* (%)	16 (0.2)	9 (0.2)	7 (0.4)	4 (0.4)
>10 ULN *n* (%)	10 (0.1)	7 (0.1)	3 (0.2)	1 (0.1)

*Notes*: *N*, number of patients with at least one post‐baseline ALT and/or AST value on treatment period. n, Number of patients with at least one event. Percentage (*n*/*N*)*100.

Abbreviations: ALT, alanine aminotransferase; AST, aspartate aminotransferase; ULN, upper limit of normal.

No hepatic failure with fatal outcome, or resulting in liver transplantation, was reported.

### 4.3 Suicidality

A total of 85 patients (1.0%) reported at least one emergent event related to suicide/self‐injury. Suicidal ideation and suicide attempt were the most commonly reported (56 patients, 0.7% and 23 patients, 0.3%, respectively) and were more frequent with the 25–50 mg dose; they were notified as serious in 80% of patients and led to treatment withdrawal in 46% of patients. The event occurred firstly within the first month of treatment for 26 out of 85 patients (30.6%) and in the second month for 17 patients (20.0%). All but one patient with suicidal ideation had relevant medical history and/or triggering factors (Table 4).

**TABLE 4:** Estimated incidence of suicidal ideation, suicide attempt and completed suicide during the treatment period

EAE	All agomelatine doses (*N* = 8467)	25 mg fixed (*N* = 6439)	25–50 mg (*N* = 2028)	Patients ≥ 65 years (*N* = 1256)	Patients < 30 years (*N* = 855)
Suicidal ideation *n* (%)	56 (0.66)	31 (0.48)	25 (1.23)	6 (0.5)	4 (0.5)
Suicide attempt *n* (%)	23 (0.27)	11 (0.17)	12 (0.59)	3 (0.2)	2 (0.2)
Completed suicide *n* (%)	2 (0.02)	1 (0.02)	1 (0.05)	‐	‐

*Note*: *n*, Number of patients with at least one EAE. ‐, no case. Percentage (*n*/*N*)*100.

Abbreviation: EAE, emergent adverse events.

Two patients completed suicide. One 30‐year‐old patient, with bipolar disorder history and 6 previous suicide attempts, treated for 18 days with the 25 mg dose, completed suicide 21 days after having decided to stop treatment. One 39‐year‐old patient with impulsive personality treated with the 50 mg dose completed suicide after 3 months of treatment. The patient had a high risk of suicide according to MINI suicidality scale at baseline. All cases of completed suicides and suicide attempts (24 cases) were reviewed by an expert: events were judged not related to the agomelatine treatment in 17 patients and doubtfully related in 7 patients. According to the expert's judgement, all these cases are particularly eloquent of the scientific literature on the topic of treatment emergence or worsening of suicidal behaviour. Whatever the delay of occurrence of the suicidal act after the initiation of the treatment, all the patients were carrying many risk factors, particularly a past history of suicide attempt, a significant suicide risk at baseline and were difficult to treat (comorbidities, lack of response to previous treatments, family conflicts). This explains why it cannot be assumed that any of the described cases may be directly attributable to the antidepressant investigated. Patients aged <30 years were not at increased suicidal risk (incidence rate: 0.7%).

### 4.4 Skin reaction

A total of 144 patients (1.7%) reported at least one EAE in the skin and subcutaneous tissue disorders (MEDRA, System Organ Class), without dose related effect (1.8% and 1.4% in 25 mg and 25/50 mg respectively). The most frequent (>10 patients) were hyperhidrosis (28 patients, 19.4%), eczema (15 patients, 10.4%), alopecia, pruritus and pruritic rash (11 patients for each, 7.6% each), except alopecia these events are already listed for agomelatine. These events led to study treatment withdrawal in 39 out of the 144 patients (27.1%; 0.5% of the whole cohort) and were considered as study treatment related in 60 patients (41.6%; 0.7% of the cohort). For most patients (81%,3%), the first event occurred within the first 3 months of treatment. All but 5 out of 164 EAEs resolved (1 with sequelae: a dermatitis) or were recovering at the cut‐off date; the 5 events not resolved were 2 eczemas, 1 acne, 1 pruritus generalised and 1 hidradenitis (Table 5).

**TABLE 5:** EAEs related to Skin and subcutaneous tissue disorders—Safety cohort and patients aged ≥ 65 years

	EAE		Severe EAE		Serious EAE		EAE leading to drug withdrawal		EAE related to study drug	
	*n*	%	*n*	%	*n*	%	*n*	%	*n*	%
Safety cohort (*N* = 8467)	144	1.7	7	0.1	10	0.1	39	0.5	60	0.7
Patients ≥ 65 years (*N* = 1256)	26	2.1	3	0.2	1	0.1	8	0.6	7	0.6

*Note*: *n*, Number of patients with at least one EAE. Percentage (*n*/*N*)*100.

Abbreviation: EAE, emergent adverse events.

Serious skin reactions were reported in 10 patients, presenting with 12 SAEs of which 2 were considered as severe (one case of cold sweats and one decubitus ulcer). All the serious skin adverse events were recovered or recovering at the end of the study.

### 4.5 Patients aged 65 years or older

The percentages of patients who reported at least one EAE was 28.7%; serious EAEs were reported in 6.8% of patients (agomelatine 25 mg: 5.9%; agomelatine 25–50 mg: 10.9%).

Among patients aged 65 years or older, 36 (2.9%) reported at least one emergent hepatic disorder. The incidence of emergent abnormal AST and/or ALT value (1%) was similar as in the whole cohort (Table 3).

Patients aged ≥65 years were not at increased suicidal risk (incidence rate: 0.7%).

Patients aged 65 years or older reported similar incidence rate of skin reactions (2.1%) with one serious, a decubitus ulcer. The first event occurred within the first 3 months of treatment (for 76.9% of patients), 3 out of 30 skin events were not recovered, 2 eczema and 1 pruritus generalised.

## 5 EFFECTIVENESS

### 5.1 Functional improvement and CGI severity of illness score

The mean CGI‐Severity illness scores decreased between baseline (median: 5, markedly ill) and the last post‐baseline assessment (mean change of −1.9 ± 1.5).

For the three SDS sub scores, patients reported less symptom‐related impairments over the treatment period in terms of work/daily activities (mean change from baseline to last visit: −3.3 ± 2.9), in social life (−3.4 ± 2.8), and in family life (−3.3 ± 2.8). Numbers of days lost and underproductive days were also diminished (mean change from baseline to last visit: −1.7 ± 2.8 and −2.6 ± 2.8, respectively; Table 6).

**TABLE 6:** SDS mean change from baseline during agomelatine treatment. Agomelatine cohort

Change between baseline and last visit on treatment period		ALL (*N* = 8453)
Total score of SDS	*n*	7338
	Mean ± SD	−9.0 ± 7.5
	Min; Max	−30; 24
Work/School	*n*	4947
	Mean ± SD	−3.3 ± 2.9
	Min; Max	−10; 10
Social life	*n*	7336
	Mean ± SD	−3.4 ± 2.8
	Min; Max	−10; 9
Family life and home responsibilities	*n*	7337
	Mean ± SD	−3.3 ± 2.8
	Min; Max	−10; 10
Days lost	*n*	7187
	Mean ± SD	−1.7 ± 2.8
	Min; Max	−7; 7
Days unproductive	*n*	7151
	Mean ± SD	−2.6 ± 2.8
	Min; Max	−7; 7

*Note*: *n*, Number of patients with an available baseline and at least one available value on treatment period.

## 6 DISCUSSION

Non‐interventional studies give the opportunity to obtain further information on a medecine's safety in a real‐life situation without selecting population as in clinical trials. The present observational study aimed at evaluating the safety of agomelatine treatment in current medical practice conditions and results confirm the known tolerability profile of this drug (de Bodinat et al., 2010; Servier Laboratories, 2015; Taylor et al., 2014). The analysed population was a large cohort of patients from six European countries (8453 patients), patients' recruitment was almost balanced between psychiatrists (60%) and general practitioners (40%); patients were markedly ill according to CGI and had a moderate functioning impairment as assessed by SDS; one‐third of patients presented a suicidal risk according to M.I.N.I. suicidality evaluation. Uptitration to 50 mg was required for 23.9% of patients, which is in line with the ratio observed in agomelatine clinical trials (15%–35%); Kennedy & Emsley, 2006). These patients had clinical characteristics of a medically complex population to treat, with a higher disability and number of previous depressive episodes and hospitalizations, and higher suicidal risk for the current episode.

The mean treatment period by agomelatine was 6 months, during which depressive symptoms improved according to CGI score, the most common evaluation used in routine clinical practice (CGI‐S, mean change of −1.9 ± 1.5). Functionality in daily life was not affected by drug acceptability as patient SDS scores improved in the 3 domains explored (work, social life, and family life & home responsibilities) and were associated with a decrease in the number of underproductive or lost days.

During the study, no unexpected EAEs were reported in agomelatine‐treated patients: the nature and frequency of adverse events reported were in accordance with previous knowledge obtained during clinical trials development and comparable to the known information on the product safety profile (Servier Laboratories, 2015). As expected, a more frequent report of serious EAEs was described for the group of patients who required uptitration to 50 mg than in patients treated with the 25 mg fixed dose, a finding likely related to the complex medical history of these patients. Similar rates of EAE were reported in the whole population and in the subset of patients aged 65 years and over. Overall, the acceptability profile of agomelatine appears distinctly better than currently available standard treatments, with discontinuations for side effects among the lowest among second‐generation antidepressants (Cipriani et al., 2018).

A focus was made on hepatic events related to the use of agomelatine owing to the drug potential to elevate liver enzymes. As regards transaminases increases (>3 ULN), a case‐under‐treatment incidence of 0.8% at 25 mg dose and 1% at 25–50 mg dose were observed. These values are comparable to those reported in the SmPC (1, 25% at 25 mg, 2, 62% at 50 mg). These transaminases elevations (AST and/or ALT ≥ 3 ULN) were in majority within the range of ]> 3–5 ULN] (54%), mainly asymptomatic, isolated and appeared mostly within the first 3 months. Unless medical explanation (e.g., NASH, known history of liver injury), most of liver function normalized within few weeks following treatment discontinuation. Two Hy's law cases (two patients with an increase in transaminases associated with cholelithiasis, both not related to agomelatine) were reported, but no liver transplant, no fulminant hepatitis, no case of hepatic failure, and no hepatic disorder with fatal outcome were observed. Whatever, in most cases, patients are clinically asymptomatic and abnormal results on liver function tests are the only elements that may raise a suspicion of antidepressant‐induced liver injury. It should be mentioned that in clinical trials, where serum transaminases are monitored regularly, the risk is adequately minimized (Perlemuter et al., 2016) with no emergence of severe symptomatic cases. As consequence, the monitoring of transaminases values required since 2012 with respect of contra indications are the most effective ways of minimising hepatic risk. In general health care, such measures are considered adapted to ensure an optimal follow‐up of depressed patients (Voican et al., 2016). A recent analysis in a cohort comprising 3.2 million new users of antidepressants has shown that, in routine clinical practice, agomelatine did not increase the risk of hospitalisation due to acute liver injury when compared to citalopram (Pladevall‐Vila et al., 2019). As risk minimisation measures (compliance with relevant contra‐indications, precautions of use, and biological liver testing before and during treatment) were in place in the populations studied, this might contribute to the lower risk found for agomelatine versus citalopram.

All antidepressant drugs may potentially cause hepatotoxic side events, even at therapeutic doses, but antidepressant‐induced hepatotoxicity has been underestimated in the scientific literature, so the evidence is insufficient for rigorous conclusions to be drawn about the prevalence and severity of these events. Several antidepressants including imipramine, amitriptyline, duloxetine, bupropion, trazodone, and agomelatine are associated with greater risks, whereas citalopram, escitalopram, paroxetine, and fluvoxamine seem to have the least potential for hepatotoxicity (Voican, Corruble, Naveau, & Perlemuter, 2014). In real‐life practice, there is no evidence of an increased risk of serious liver injury following initiation of SNRIs (venlafaxine, milnacipran, duloxetine) or other antidepressants (mianserin, mirtazapine, tianeptine and agomelatine) compared with SSRIs (fluoxetine, citalopram, paroxetine, sertraline, fluvoxamine, and escitalopram; Billioti de et al., 2018).

A thorough evaluation of the patient's suicide risk has been performed during the study. The incidence did not differ according to the age of the patients (0.7% for patients aged <30 years or ≥65 years vs. 1.0% for patients aged <65 years) but was numerically more elevated in the group of patients who required uptitration to 50 mg (1.5% vs. 0.7% at 25 mg), a difference that can be due to more severe depression characteristics at the start of treatment in the 25–50 mg group. The two completed suicides were not considered related to agomelatine.

In general, it is assumed that antidepressants are beneficial for all symptoms of depression, including suicidality. Some evidence suggests that antidepressant, including SSRIs, may cause worsening of suicidal ideas in vulnerable patients, though systematic reviews and pooled analysis of experimental, observational, and epidemiological studies failed to provide a clear relationship between their use and increased suicidal ideation and behaviour in adults (Nischal, Tripathi, Nischal, & Trivedi, 2012). For all antidepressants, this risk can be anticipated and managed clinically and there is a need for early follow‐up and encouraging support and supervision of patients, especially in the early phase of treatment.

The occurrence of cutaneous events was mentioned in the SmPC at the time of agomelatine launch. Results of the present cohort confirm that the frequency of these events did not differ according to the dose prescribed (1.4% at 25–50 mg vs. 1.8% at 25 mg) or the age of patients (2.1%, ≥65 years). Based on these cohort data, the European Medical Agency (EMA) decided not to consider any more skin events as a potential risk for agomelatine.

Cutaneous side effects have been described with all psychotropic drugs; they are variable and numerous and usually benign, but may occasionally carry significant morbidity (Bliss & Warnock, 2013; Mitkov, Trowbridge, Lockshin, & Caplan, 2014).

The sustained adherence observed in this cohort study is in line with the recommendations supporting duration of at least 6 months of the treatment period to achieve a sufficient improvement of symptoms and prevent the chronic evolution of the disease (ANAES, 2002; Davidson, 2010; Mitchell, 2006; Qaseem, Snow, Denberg, Forciea, & Owens, 2008; Wade, Despiegel, & Heldbo, 2006). It may be hypothesized that the level of adherence and the observed efficacy of the agomelatine treatment are directly linked to the good acceptability of the treatment by patients, hypothesis supported by a low rate of treatment discontinuation due to AE in the study.

A first limitation of this prospective study was that physicians who accepted the study, compared to those who never answered or refused, were probably more prone to work in accordance with recommendations of treatment for depression or product SmPC. Second, it cannot be ruled out that the duration of exposure has been inflated by the recruitment of patients who were more compliant or more concerned by the disease, and as such might more easily adhere to the study medication. Finally, as a result of the non‐interventional design, investigations were limited, thus no detailed information was collected regarding the evolution of the different symptoms associated with depression.

The results of this international, observational, prospective, non‐interventional study confirm under daily practice conditions the safety profile of agomelatine given at the therapeutic recommended doses. While patients' functioning and symptomatic distress were improved by the treatment, collected safety data did not reveal any new risk compared to those described in the SmPC and allowed a more accurate assessment of potential risks in usual practice. These findings obtained in a large representative population of out‐patients suffering from MDD confirm the favourable tolerability profile of agomelatine.

## CONFLICT OF INTERESTS

Philip Gorwood received during the last 5 years fees for presentations at congresses or participation in scientific boards from Alcediag‐Alcen, Angelini, GSK, Janssen, Lundbeck, Otsuka, SAGE and Servier; Jacques Benichou received during the last 10 years fees for participation in scientific boards from Astra Zeneca, Pierre Fabre, IRIS and Research Triangle Institute; Nicholas Moore has received Honoraria for Servier study scientific committee; Enric Álvarez Martínez has received consulting and educational honoraria from Eli Lilly, Lundbeck, Pfizer, Sanofi‐Aventis and Otsuka; has participated as principal local investigator in clinical trials sponsored by Eli Lilly, Bristol‐Myrers Squibb, Sanofi‐Aventis and AB·Biotics; and has served as national coordinator of clinical trials sponsored by Servier and Lundbeck. Joost Mertens declared no conflict of interest, Eugenio Aguglia has received during the last 2 years, fees participation in scientific boards from Angelini, Glaxo, Lundbeck, Luisa Figueira has received consulting and educational honoraria from Eli Lilly, Lundbeck, Pfizer, Servier and Astra‐Zéneca; has participated as principal local investigator in clinical trials sponsored by Eli Lilly, Servier, Astra‐Zéneca Janssen; and has served as national coordinator of clinical trials sponsored by Servier and Eli Lilly, Peter Falkai has received research support and honoraria for lectures or advisory activities from Abbott, Janssen, Lundbeck, Otsuka, Recordati, Richter, Servier and Takeda. Valérie Olivier, Marine Wattez, Françoise Picarel‐Blanchot and Christian de Bodinat are employees at Servier. This study was sponsored by Servier (Suresnes, France).
